# Quantitative flow ratio-guided versus angiography-guided operation for valve disease accompanying coronary heart disease

**DOI:** 10.3389/fcvm.2023.1076049

**Published:** 2023-03-03

**Authors:** Wenlong Yan, Yangyang Wang, Xin Zheng, Pengfei Guo, Sumin Yang

**Affiliations:** ^1^ Department of Cardiovascular Surgery, The Affiliated Hospital of Qingdao University, Qingdao University, Qingdao, China; ^2^ Department of Nuclear Medicine, The Affiliated Hospital of Qingdao University, Qingdao University, Qingdao, China; ^3^ Surgical Operating Room, The Affiliated Hospital of Qingdao University, Qingdao University, Qingdao, China

**Keywords:** angiography, quantitative flow ratio, coronary artery bypass, valve replacement, physiological assessment

## Abstract

**Background:**

Valve replacement combined with coronary artery bypass graft (CABG) operation (VR + CABG) is usually associated with higher mortality and complication rates. Currently, angiography remains the most commonly used approach to guide CABG. The aim of this study is to investigate whether a quantitative flow ratio (QFR)-guided strategy can improve the clinical outcomes of VR + CABG.

**Methods:**

Patients (*n* = 536) treated by VR + CABG between January 2018 and December 2021 were retrospectively assessed. In 116 patients, all lesions were revascularized entirely based on QFR (the QFR-guided group), whereas in 420 patients, all lesions were revascularized entirely based on angiography (the angiography-guided group). To minimize selection bias between the 2 groups, propensity score matching was performed at a ratio of 1:2. The primary endpoint of the study was the rate of major adverse cardiac and cerebrovascular events (MACCE) at 1-year, which was defined as a composite of cardiac mortality, myocardial infarction (MI), any repeat revascularization, and stroke.

**Results:**

No statistically significant differences were observed in the baseline clinical characteristics between the QFR-guided and angiography-guided groups after propensity score matching. The mean age of all patients was 66.2 years [standard deviation (SD) = 8.3], 370 (69%) were men, the mean body-mass index of the population was 24.8 kg/m^2^ (SD = 4.5), 129 (24%) had diabetes, and 229 (43%) had angina symptoms. When compared with the angiography-guided group, the QFR-guided group had a significantly shorter operative time (323 ± 60 min vs. 343 ± 71 min, *P* = 0.010), extra corporal circulation time (137 ± 38 min vs. 155 ± 62 min, *P *= 0.004), clamp time (73 ± 19 min vs. 81 ± 18 min, *P* < 0.001), and less intraoperative bleeding volume (640 ± 148 ml vs. 682 ± 166 ml, *P* = 0.022). Compared with the angiography-guided group, the 1-year MACCE was significantly lower in the QFR-guided group (6.9% vs. 14.7%, *P* = 0.036, hazard ratio = 0.455, 95% confidence interval: 0.211–0.982).

**Conclusion:**

Our results raise the hypothesis that among patients who undergo VR + CABG, QFR-guided strategy is associated with optimized surgical procedure and a superior clinical outcome, as evidenced by a lower rate of MACCE at 1-year compared with conventional angiography-guided strategy.

## 1. Introduction

Valve combined coronary artery bypass graft (CABG) operation still accounts for a significant proportion of adult cardiac surgery according to the Society of Thoracic Surgeons (STS) Adult Cardiac Surgery Database (ACSD) in 2021 (1). Particularly, patients receiving valve replacement (VR) + CABG exhibit clinical features that generally place them at a higher risk than patients receiving valve operations or CABG alone. Whether or which lesions should be revascularized intraoperatively remains controversial for such patients. Presently, visual assessment based on coronary angiography is the main method to guide CABG, however, coronary angiography can only identify lesions with anatomic narrow and cannot assess the physiological impact of lesions on the myocardium they dominate. The physiological assessment of coronary artery has been recommended to evaluate the severity of coronary stenosis (2). These recommendations are primarily based on the excellent performance of fractional flow reserve (FFR) in randomized controlled trials guiding percutaneous coronary intervention (PCI) (3–5). Although some relevant guidelines have strongly recommended the use of physiological assessment based on pressure wires to assess moderate stenosis (2), it is largely underutilized in practice because of the long operation time, potential complications of pressure wires, and the side-effects of pharmacological agents. The calculation of quantitative flow ratio (QFR) is mainly based on three-dimensional (3D) coronary models derived from invasive coronary angiography and fast computational fluid dynamics, which enables the online FFR estimation without using any pressure guidewire and vasodilator drugs (6, 7). Previous studies have demonstrated that online QFR have satisfactory feasibility and accuracy in evaluating the hemodynamics of vessel stenosis when compared with FFR (8, 9). Therefore, in this study, we investigated whether a QFR-guided lesions selection strategy can improve the clinical outcomes of VR + CABG.

## 2. Methods

### 2.1. Study design

**Figure 1:**
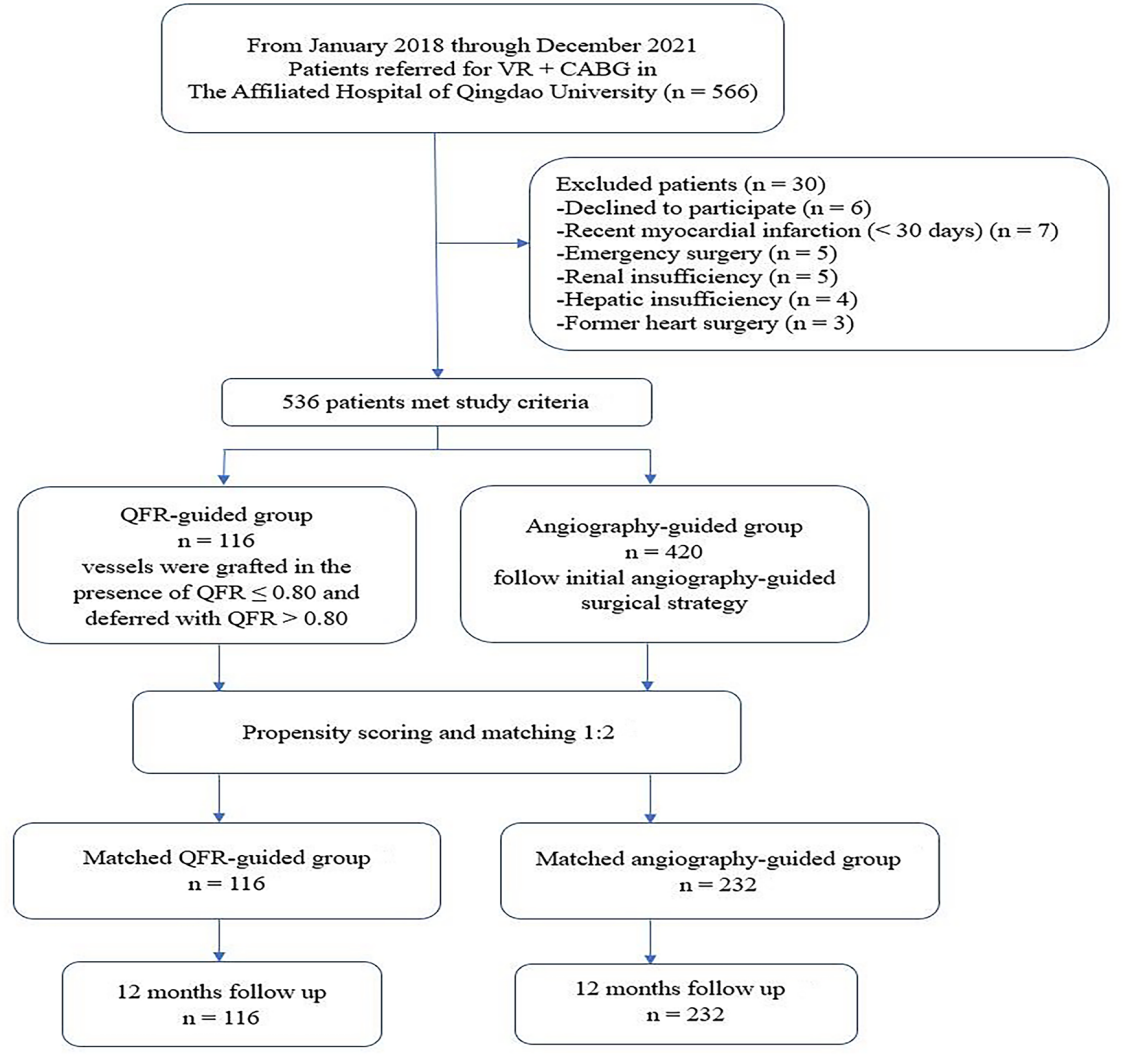
Study flow chart. VR + CABG, heart valve surgical replacement combined with coronary artery bypass graft; QFR, quantitative flow ratio.

We retrospectively analyzed adults (≥18 years, *n* = 566) who underwent VR + CABG at the Affiliated Hospital of Qingdao University from January 2018 to December 2021 (Figure 1). Patients (*n* = 6) who declined to participate and those (*n* = 24) with emergency operation, renal insufficiency, hepatic insufficiency, recent myocardial infarction (MI) (<30 days), or former heart surgery were all excluded. The included population was then assigned to QFR-guided or angiography-guided groups. The patients (*n* = 116) were assigned to the QFR-guided group if all lesions of QFR ≤ 0.8 were revascularized and QFR > 0.8 were deferred. The patients (*n* = 420) were assigned to the angiography-guided group if all lesions were revascularized entirely based on angiography.

The present study was approved by the local ethics committee, and the requirement of individual consent for this retrospective analysis was waived.

### 2.2. Coronary angiography

The puncture point for coronary angiography was the radial or femoral artery, the diameter of the puncture sheath was 5-F or 6-F, and the images were obtained using the x-ray systems (Allura X per FD20, Philips; Innova IGS520, GE) at ≥15 frames/s. The preoperative anticoagulation strategy for coronary angiography was intravenous heparin at 100 IU/kg.

### 2.3. QFR analysis

QFR was performed on any lesions with a visual reference vessel ≥1.5 mm in diameter. For each lesion, at least two angiographic images with a difference of >25° in the projection angle were transmitted to the Angio Plus system (Pulse Medical Imaging Technology, Shanghai, China) for QFR calculation. The analyst manually placed markers at the proximal and distal locations of the detected vessel, and the system automatically outlined the contours of the detected vessel. If the traced vessel trajectory deviated from the normal lumen, additional markers were placed, or the vessel outline was manually edited. The quantitative coronary angiography mainly reported reference vessel diameters, minimal lumen diameter, minimal lumen area, and percent diameter/area stenosis. An artificial intelligence-assisted computing software (Angio Plus Core, Pulse Medical Imaging Technology, Shanghai, China) combined vascular image information from multiple angles with estimated vessel flow to obtain a 3D-QFR. QFR evaluation was performed later off-line by a blinded core laboratory (Pulse Medical Imaging Technology, Shanghai, China). Each patient's QFR was independently interpreted by two observers. The observers were blinded to all clinical information except for the diagnosis. If the QFR conclusions of these two observers were inconsistent, a third observer participated.

### 2.4. Surgical technique

The operation type, graft type, and specific procedures were conducted at the discretion of the surgeon. The grafts used for CABG included the internal mammary artery, radial artery, and saphenous vein. The replacement valves were Carpentier-Edwards PERIMOUNT Plus Pericardial Bioprosthesis (Edwards Lifesciences, Irving, CA) and St. Jude Medical Regent Mechanical Heart Valve (St. Paul, MN).

### 2.5. Study endpoints

The primary endpoint of the study was the rate of major adverse cardiac and cerebrovascular events (MACCE) at 1-year postoperatively, which was defined as a composite of cardiac mortality, MI, any repeat revascularization, and stroke. Secondary endpoints were cardiac mortality, MI, any repeat revascularization, stroke, worsening in the NYHA class of ≥1, rehospitalization for heart failure and valve reoperation at 1-year postoperatively. MI was defined as described previously (10).

### 2.6. Sample size and power calculation

The primary purpose of this study was to assess MACCE after 1 year in patients who underwent QFR-guided VR + CABG versus angiography-guided VR + CABG. We estimated the MACCE rate of 6.1% after 1 year in the QFR-guided group, as well as a MACCE rate of 15.2% after 1 year in the angiography-guided group. These rates of MACCE were based on the data from the study by Bowdish et al. (11) and the results of our center. We estimated a minimum sample size of 116 patients in the QFR-guided group and 232 in the angiography-guided group, based on a 2-sided Chi-square test with an alpha level of 0.05 and a statistical power of 0.80.

### 2.7. Statistical analysis

All statistical analyses were performed using the R version 4.2.1, and the two-tailed probability values <0.05 were considered statistically significant. Continuous variables with a normal distribution were described as the mean ± standard deviation (SD), and differences between these variables were compared by Student's *t*-test. The median and interquartile ranges were calculated to describe the continuous variables that did not conform to a normal distribution, and the differences between these variables were compared by the Mann–Whitney *U* test. Categorical variables were described as frequencies and percentages, and the differences between these variables were compared by the Pearson's *χ*^2^ test or Fisher's exact test. MACCE and its constituent events were compared by Cox proportional hazards analysis, and Kaplan–Meier analysis was performed to present the primary and secondary endpoints at 1-year postoperatively. To minimize any bias between the 2 groups, propensity score matching at a ratio of 1:2 was utilized to compare clinical outcomes from patients in the 2 groups. The patients in the QFR-guided group were matched to the angiography-guided group by all the preoperative variables in the Table 1. The nearest neighbor method was applied with a caliper of 0.2, and the balance after matching was evaluated with standardized mean differences (SMD).

**Table 1:** Baseline characteristics of the study population pre-PSM.

	QFR-guided (*n* = 116)	Angio-guided (*n* = 420)	*P* value
Age (years)	67.1 ± 8.0	65.3 ± 7.8	0.029
Male	85 (73%)	285 (68%)	0.264
BMI (kg/m^2^)	25.0 ± 4.5	24.6 ± 4.3	0.380
Hypertension	40 (34%)	168 (40%)	0.280
Hypercholesterolemia	35 (30%)	138 (33%)	0.584
Diabetes mellitus	31 (27%)	98 (23%)	0.421
Previous MI	20 (17%)	63 (15%)	0.555
Smoking history	58 (50%)	193 (46%)	0.439
Cerebrovascular diseases	9 (7.8%)	28 (6.7%)	0.681
ACS	6 (5.2%)	19 (4.5%)	0.769
CCS classification			0.704
No angina	71 (61%)	236 (56%)	
I	19 (16%)	84 (20%)	
II	22 (19%)	75 (18%)	
III	3 (3%)	21 (5%)	
IV	1 (1%)	4 (1%)	
LVEF, %			0.786
<35	6 (5%)	25 (6%)	
35–50	29 (25%)	116 (28%)	
>50	81 (70%)	279 (66%)	
LVEDD, mm	52.5 ± 7.26	51.6 ± 8.14	0.282
SYNTAX score	24.4 ± 7.66	24.7 ± 7.91	0.716
Valve disease type			0.740
Aortic valve disease	61 (53%)	235 (56%)	
Mitral valve disease	41 (35%)	143 (34%)	
Aortic + mitral valve disease	14 (12%)	42 (10%)	

BMI, body mass index; MI, myocardial infarction; ACS, acute coronary syndrome; CCS, Canadian Cardiovascular Society; LVEF, left ventricular ejection fraction; LVEDD, left ventricular end-diastolic diameter.

## 3. Results

### 3.1. Baseline characteristics

**Figure 2:**
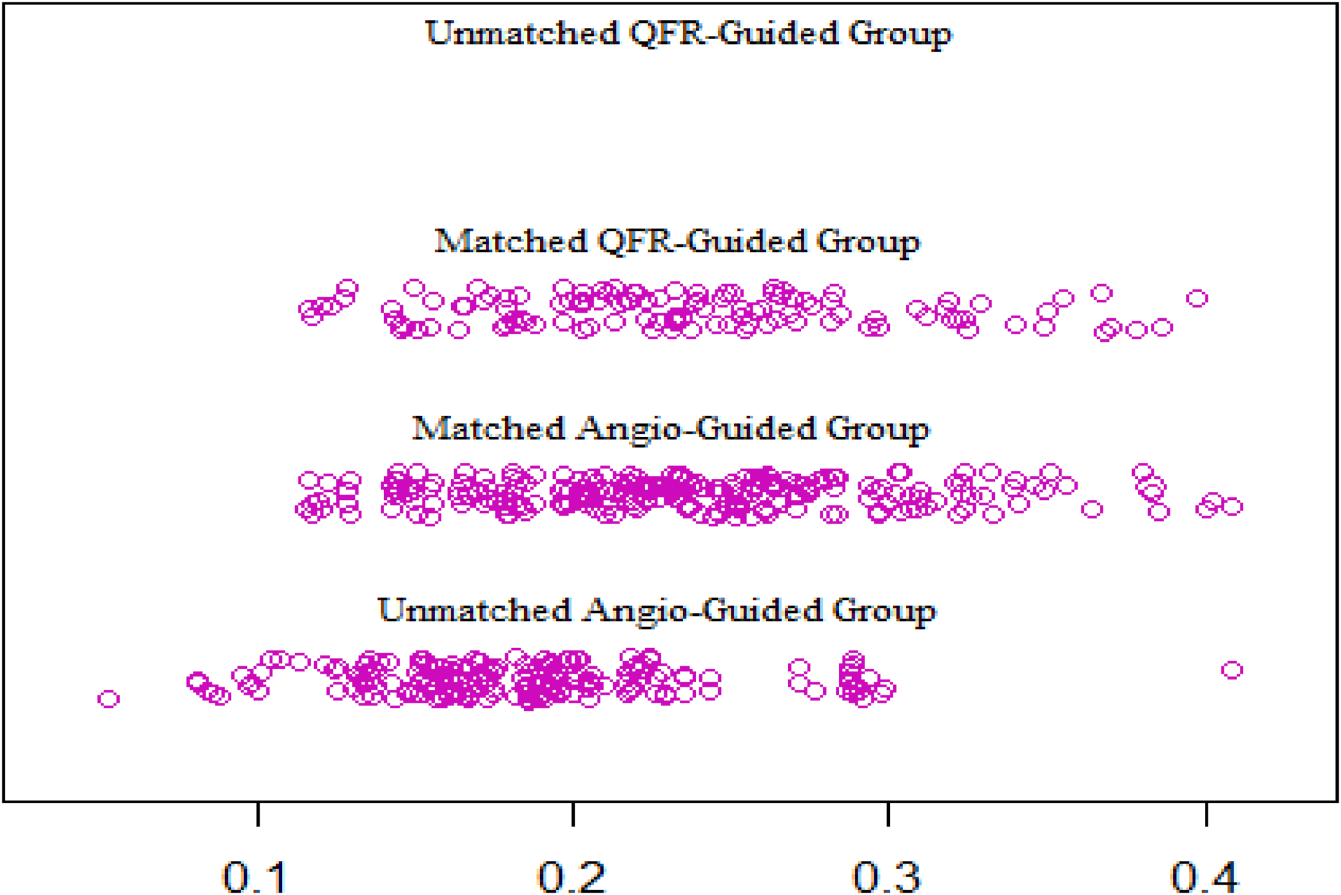
Distribution of the propensity scores.

From January 2018 to December 2021, a total of 536 patients’ clinical data were collected, of which 116 patients fully met the QFR guidance criteria and were included in the QFR guidance group and 420 patients were included in the angiography guidance group. The details about the clinical characteristics of the patients are summarized in Table 1. There was statistically significant difference in the ages between the two groups. The baseline differences were balanced by propensity matching between the two groups (Table 2) and the distribution of propensity scores is presented in Figure 2. The mean age of all patients was 66.2 years (SD: 8.3) and 370 (69%) were men. The mean BMI of the population was 24.8 kg/m^2^ (SD: 4.5), 129 (24%) had diabetes, and 229 (43%) exhibited angina symptoms.

**Table 2:** Baseline characteristics of the study population post-PSM.

	QFR-guided (*n* = 116)	Angio-guided (*n* = 232)	*P* value	SMD
Age (years)	67.1 ± 8.0	67.5 ± 7.7	0.652	−0.057
Male	85 (73%)	175 (75%)	0.663	−0.049
BMI (kg/m^2^)	25.0 ± 4.5	24.7 ± 4.6	0.564	0.051
Hypertension	40 (34%)	88 (38%)	0.529	−0.082
Hypercholesterolemia	35 (30%)	69 (30%)	0.934	0.009
Diabetes mellitus	31 (27%)	62 (27%)	1.000	0.000
Previous MI	20 (17%)	35 (15%)	0.603	0.057
Smoking history	58 (50%)	113 (49%)	0.820	0.026
Cerebrovascular diseases	9 (7.8%)	22 (9%)	0.595	−0.064
ACS	6 (5.2%)	11 (4.7%)	0.861	0.030
CCS classification			0.964	0.037
No angina	71 (61%)	139 (60%)		
I	19 (16%)	39 (17%)		
II	22 (19%)	42 (18%)		
III	3 (3%)	10 (4%)		
IV	1 (1%)	2 (1%)		
LVEF, %			0.973	0.045
<35	6 (5%)	11 (5%)		
35–50	29 (25%)	60 (26%)		
>50	81 (70%)	161 (69%)		
LVEDD, mm	52.5 ± 7.26	52.4 ± 7.42	0.905	0.003
SYNTAX score	24.4 ± 7.66	24.1 ± 7.75	0.733	0.043
Valve disease type			0.926	0.018
Aortic valve disease	61 (53%)	127 (55%)		
Mitral valve disease	41 (35%)	79 (34%)		
Aortic + mitral valve disease	14 (12%)	26 (11%)		

BMI, body mass index; MI, myocardial infarction; ACS, acute coronary syndrome; CCS, Canadian Cardiovascular Society; LVEF, left ventricular ejection fraction; LVEDD, left ventricular end-diastolic diameter.

### 3.2. Operative characteristics

The operative outcomes of the patients are summarized in Table 3. Of 348 patients, 188 (54.0%) underwent mitral VR + CABG (MVR + CABG), 120 (34.5%) underwent aortic VR + CABG (AVR + CABG), and 40 (11.5%) underwent MVR + AVR + CABG (DVR + CABG). The number of anastomoses per patient was statistically different between the two groups (1.8 ± 0.9 vs. 2.1 ± 1.2, *P* = 0.018). When compared with the angiography-guided group, the QFR-guided group showed a significantly shorter operative time (323 ± 60 min vs. 343 ± 71 min, *P* = 0.010), extra corporal circulation time (137 ± 38 min vs. 155 ± 62 min, *P* = 0.004), clamp time (73 ± 19 min vs. 81 ± 18 min, *P* < 0.001), and less intraoperative bleeding volume (640 ± 148 ml vs. 682 ± 166 ml, *P* = 0.022).

**Table 3:** Operative outcomes post-PSM.

	QFR-guided (*n* = 116)	Angio-guided (*n* = 232)	*P* value
Operation methods			0.956
MVR + CABG	61 (53%)	127 (55%)	
AVR + CABG	41 (35%)	79 (34%)	
DVR + CABG	14 (12%)	26 (11%)	
Anastomoses per patient	1.8 ± 0.9	2.1 ± 1.2	0.018
Grafted coronary arteries			0.389
LAD grafted	78 (37%)	139 (33%)	
Diagonals grafted	25 (12%)	63 (15%)	
CX grafted	50 (24%)	88 (21%)	
RCA grafted	56 (27%)	130 (31%)	
Operative time, min	323 ± 60	343 ± 71	0.010
ECC time, min	137 ± 38	155 ± 62	0.004
Clamp time, min	73 ± 19	81 ± 18	<0.001
Hospital stay time, day	24 ± 5.0	25 ± 6.1	0.128
RBC transfusion, units	2.6 ± 1.8	2.8 ± 1.7	0.311
Intraoperative bleeding volume	640 ± 148	682 ± 166	0.022

MVR, mitral valve replacement; AVR, aortic valve replacement; DVR, double valve replacement; CABG, coronary artery bypass graft; LAD, left anterior descending coronary artery; CX, circumflex coronary artery; RCA, right coronary artery; ECC, extra corporal circulation; RBC, red blood cells.

### 3.3. Clinical outcomes

**Figure 3:**
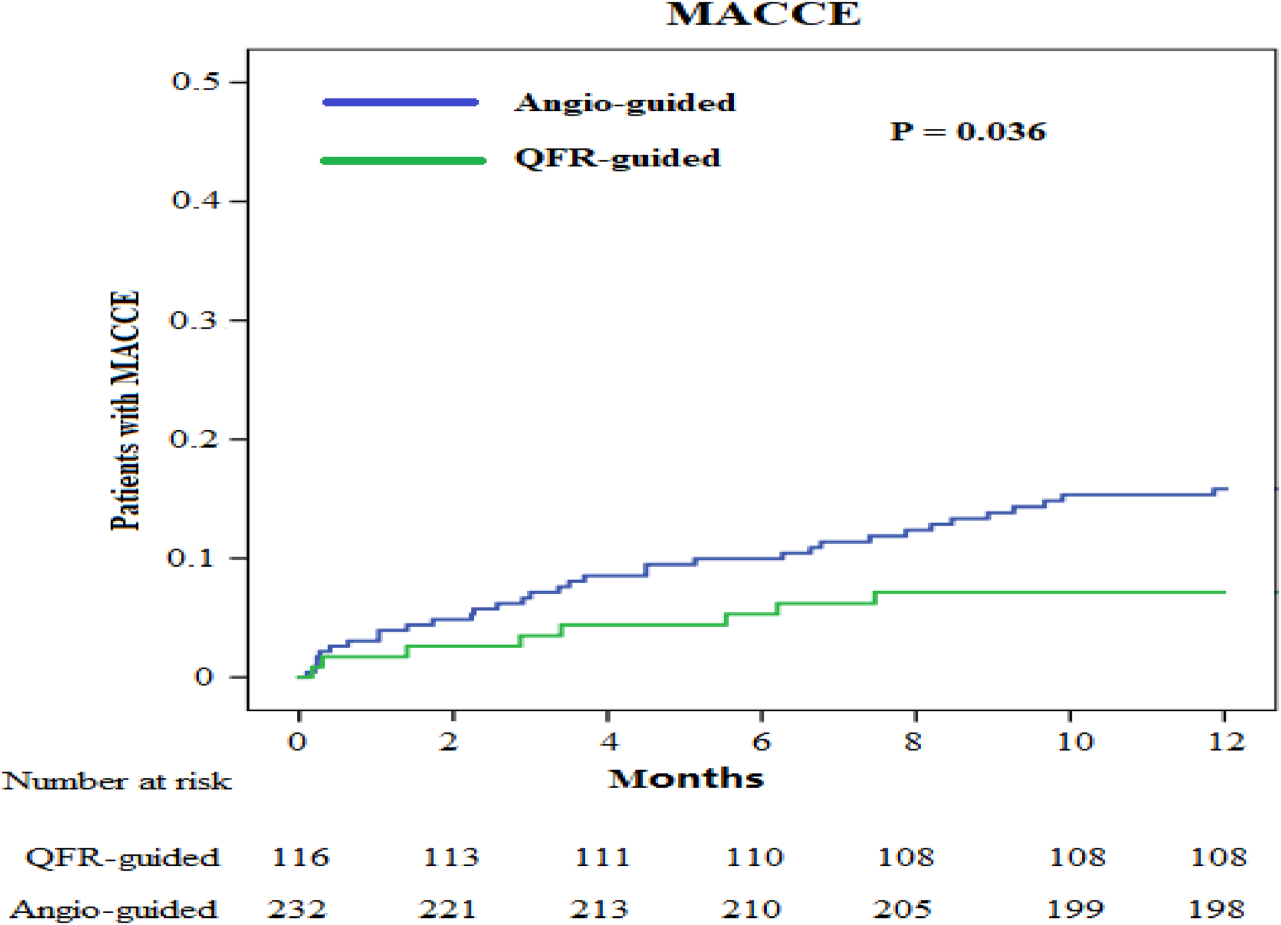
Kaplan–Meier curves for the primary endpoint.

**Figure 4:**
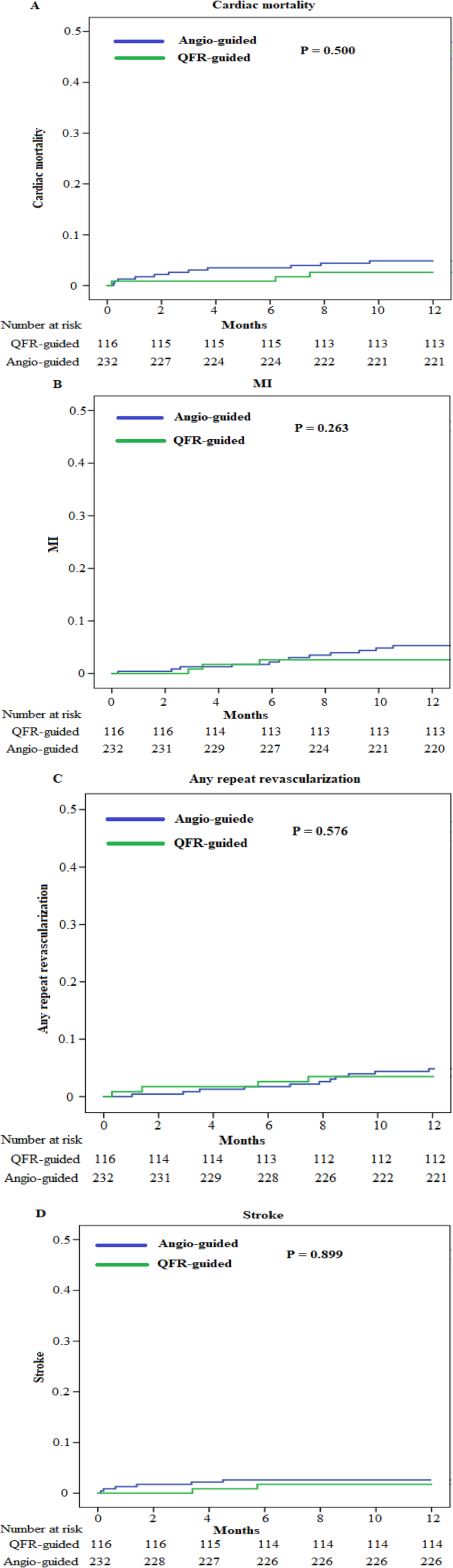
Kaplan–Meier curves for the secondary endpoints. (**A**) Kaplan–Meier curves for cardiac mortality. (**B**) Kaplan–Meier curves for MI. (**C**) Kaplan–Meier curves for any repeat revascularization. (**D**) Kaplan–Meier curves for stroke.

We obtained the clinical outcome data for all patients *via* outpatient and telephonic follow-up (Table 4, Figures 3, 4). The composite primary endpoint occurred within 1 year in 8 of the 116 patients in the QFR-guided group and in 34 of the 232 patients in the angiography-guided group (6.9% vs. 14.7%, *P* = 0.036, HR = 0.455, 95% CI [0.211–0.982]). Kaplan-Meier analysis (Figure 3) showed that MACCE was significantly lower in the QFR-guided group than in the angiography-guided group. There were no significant difference between the QFR-guided group and the angiography-guided group in terms of the rates of cardiac mortality (2.6% vs. 4.7%, *P* = 0.500, HR = 0.530, 95% CI [0.148–1.898]), MI (2.6% vs. 5.2%, *P* = 0.263, HR = 0.477, 95% CI [0.135–1.690]), any repeat revascularization (3.4% vs. 4.7%, *P* = 0.576, HR = 0.694, 95% CI [0.211–2.181]), stroke (1.7% vs. 2.6%, *P* = 0.899, HR = 0.650, 95% CI [0.131–3.221]), worsening in NYHA class of ≥1 (4.3% vs. 4.7%, *P* = 0.856, HR = 0.915, 95% CI [0.318–2.634]), rehospitalization for heart failure (9.5% vs. 10.3%, *P* = 0.801, HR = 0.917, 95% CI [0.449–1.871]), and valve reoperation (2.6% vs. 2.2%, *P* = 0.899, HR = 1.203, 95% CI [0.288–5.035]).

**Table 4:** Clinical end points at 1-year.

	QFR-guided (*n* = 116)	Angio-guided (*n* = 232)	*P* value	HR	95% CI
Cardiac mortality	3 (2.6%)	11 (4.7%)	0.500	0.530	0.148–1.898
Myocardial infarction	3 (2.6%)	12 (5.2%)	0.263	0.477	0.135–1.690
Any repeat revascularization	4 (3.4%)	11 (4.7%)	0.576	0.694	0.211–2.181
Stroke	2 (1.7%)	6 (2.6%)	0.899	0.650	0.131–3.221
MACCE	8 (6.9%)	34 (14.7%)	0.036	0.455	0.211–0.982
Worsening in NYHA class of ≥1	5 (4.3%)	11 (4.7%)	0.856	0.915	0.318–2.634
Rehospitalization for heart failure	11 (9.5%)	24 (10.3%)	0.801	0.917	0.449–1.871
Valve reoperation	3 (2.6%)	5 (2.2%)	0.899	1.203	0.288–5.035

MACCE, major adverse cardiac and cerebrovascular events; NYHA, New York Heart Association.

## 4. Discussion

QFR is a new method for estimating FFR, which uses 3D coronary artery reconstruction and computational fluid dynamics from angiography, and reflects the ratio of coronary pressure distal to the stenosis to aortic pressure under the condition of maximal myocardial hyperemia (6, 9). Recent studies have demonstrated that physiology assessment-guided lesion selection strategy improve the clinical outcomes when compared with angiography-guided strategy in patients with coronary artery disease undergoing PCI or CABG (12–15). Valve combined with CABG operation is usually associated with a higher mortality and complication rates, and the prognosis of patients is worse than that of patients undergoing valve or CABG operation alone (16, 17). This is the first study to report that QFR-guided VR + CABG reduced MACCE at 1-year significantly and optimized the surgical procedure compared with conventional angiography-guided strategy.

QFR assessment was performed on all lesions with a visual reference vessel diameter ≥1.5 mm. Notably, angiographic and hemodynamic assessments were inconsistent in more than one-third of the patients with intermediate coronary lesions (18). In our study, this difference resulted in less average number of anastomoses in the QFR-guided group than in the angiography group (1.8 ± 0.9 vs. 2.1 ± 1.2, *P* = 0.018). This result is consistent with those of most of the previous studies (12, 19). At the same time, we observed that the QFR-guided group had shorter operative time, extra corporal circulation (ECC) time and clamp time when compared with the angiography-guided group. This observation may be related to the following results: first, the QFR-guided surgical strategy reduced the average number of anastomoses, thereby simplifying the surgical procedure and shortening the related time. Second, our surgical procedure was CABG followed by VR operation, and functional complete revascularization guided by QFR may be more accurate (20), with myocardial cardioplegia perfusion through the bridging vessels, and a shorter time to induce cardiac arrest. Third, functionally complete revascularization leads to better intraoperative myocardial protection and less myocardial damage; therefore, cardiac resuscitation is smooth and the time is short (21).

The practical implication is that QFR can identify lesions that require revascularization and those that can be safely delayed, thereby reducing the incidence of early and late myocardial infarction without increasing ischemia-driven revascularization procedures during the 1-year follow-up compared with that in angiography-guided lesion selection. Other studies have also confirmed that the patency of bypass grafts with functional revascularization is significantly higher than that of bypass grafts with non-functional revascularization (22, 23). In our study, MI and repeat revascularization were lower in the QFR-guided group than in the angiography-guided group, albeit there was no significant difference between the two groups. The possible reason for this is that our follow-up time was short. In both the groups, the increased rates of MI and repeat revascularization could be observed in the later follow-up period, which may be related to our selection of graft materials. The preferred strategy involved the routine use of the left internal mammary artery to the left anterior descending coronary artery and segments of the saphenous vein to the remaining coronary arteries requiring revascularization; therefore, the saphenous vein accounts for a relatively high proportion, resulting in: first, the biological characteristics of saphenous vein promote a high bridging vessel occlusion rate. With the extension of the follow-up time, the bridging vessels related to meaningless revascularization can result in occlusion. Second, saphenous vein anastomosed to the coronary artery with functionally insignificant stenoses might accelerate the atherogenesis process of the native vessels. These two points may have led the increased rate of late MI and repeat revascularization in our study. Moreover, we analyzed that the average number of anastomoses in the angiography-guided group was higher than that in the QFR-guided group, but the primary endpoint of MACCE was significantly lower in the QFR-guided group (6.9% vs. 14.7%, *P* = 0.036, HR = 0.455, 95% CI [0.211–0.982]). The specific reasons for this warrant further analyses. Based on the present results, the possible reason for this could be that the angiography-guided group performed more meaningless revascularization, which did not bring benefits to the patients during operation. However, increased operative time, ECC time, and clamp time may increase the perioperative cardiac mortality and stroke.

## 5. Limitation

The study has some limitations that must be acknowledged. The main limitation of the study was its retrospective and observational design; therefore, we cannot rule out selection bias, confounding of indications, and underreporting of events. Second, the accuracy of QFR measurement depends on the technique and quality of angiographic acquisition, and retrospective studies cannot control the quality of angiography. Next-generation QFR systems will require only a single projection and incorporate more automated processes, which will further reduce the analysis variability and time expenditure (24). Third, the follow-up time was relatively short.

## 6. Conclusion

The present results raise a hypothesis that among patients who undergo VR + CABG, QFR-guided strategy is associated with a lower rate of MACCE after 1 year when compared to conventional angiography-guided strategy. Meanwhile, QFR-guided strategy can optimize the operative procedure, including reducing the operative time, extra corporal circulation time, clamp time and intraoperative bleeding volume. Further studies of high-quality randomized controlled trials with larger sample size and long-term follow-up are needed.

## Data Availability

The original contributions presented in the study are included in the article/Supplementary Material, further inquiries can be directed to the corresponding author.
